# Testes mass in the livebearing fish *Brachyrhaphis rhabdophora* (Poeciliidae) varies hypoallometrically with body size but not between predation environments

**DOI:** 10.1002/ece3.4618

**Published:** 2018-11-08

**Authors:** Haley N. Brown, Brittany Herrod Gale, Jerald B. Johnson, Mark C. Belk

**Affiliations:** ^1^ Evolutionary Ecology Laboratories, Department of Biology Brigham Young University Provo Utah; ^2^ Monte L. Bean Life Science Museum Provo Utah

**Keywords:** body size, *Brachyrhaphis rhabdophora*, gonadosomatic index, life history, Poeciliidae, predation environment effects

## Abstract

In this study, we considered potential causes of variation in testis size in the livebearing fish *Brachyrhaphis rhabdophora*. We evaluated variation in testes mass among individual males and among populations that occupy different selective environments. First, we predicted that small males should allocate more to testes mass than large males (i.e., hypoallometric pattern) based on a sperm competition argument. Second, based on life history theory and associated differences in mortality rates between populations that coexist with many fish predators and those with few predators, we predicted that males in high‐predation environments should allocate more to testes mass than males in habitats with few predators. Our results showed that small males allocated proportionally more to testes mass than larger males (slope of testes mass to body mass was hypoallometric). However, there was no effect of predator environment on testes mass independent of body size differences. In this system, size‐specific patterns of reproductive allocation in males (hypoallometry) differ from that seen in females (hyperallometry). Allocation to testes mass may respond to differences in mortality rate through selection on body size.

## 1 INTRODUCTION

Reproductive allocation is a measure of how an organism allocates its time and energy to reproduction. Male reproductive allocation should be subject to many of the same selective forces and constraints that operate on female reproductive allocation, such as extrinsic mortality, resource availability, and local mate competition (Johnson, 2001; Johnson & Basolo, 2003; Law, 1979; Schradin et al., 2010; Ziege et al., 2009). Female reproductive allocation is relatively easy to quantify because it can be summarized in measures of clutch size or brood mass (Ghalambor, Reznick, & Walker, 2004). However, patterns of reproductive allocation in females do not necessarily predict patterns of reproductive allocation in males (Smith & Belk, 2018; Smith, Creighton, & Belk, 2015). Male reproductive allocation is more complex and comprises multiple factors, including display rate, investment in pigmentation, and activity rate, in addition to the more intuitive measure of testes mass (Gale, Johnson, Schaalje, & Belk, 2013; Godin, 1995; Kawase, Hayashi, Matsumoto, & Takegaki, 2017; Money, Ingley, & Johnson, 2017). Previous studies have revealed varied allometric relationships between testes size and body size, from hypoallometry (Vrech, Olivero, Mattoni, & Peretti, 2014) to isometry (Breed & Taylor, 2000; Fitzpatrick, Desjardins, Stiver, Montgomerie, & Balshine, 2006; Hettyey, Vági, Török, & Hoi, 2012) and hyperallometry (Fitzpatrick et al., 2006; Riesch et al., 2013; Vrech et al., 2014). Here, we examine how testes mass varies among males of different sizes from contrasting selective environments that vary in mortality risk (see Riesch et al., 2013).

Sperm competition theory suggests that male condition and male mating strategies could affect male reproductive investment (Parker, 1970; Stockley, Gage, Parker, & Moller, 1997). For example, in systems where females mate with multiple males, and where the volume of sperm transferred determines male mating success, theory predicts that smaller males that act as sneakers (or satellites) should invest more in testes mass than larger males that primarily use display tactics (Hankison & Ptacek, 2007; Kawase et al., 2017; Neff, Fu, & Gross, 2003). This is because individual reproductive attempts using sneak‐and‐thrust tactics are less likely to result in successful fertilization than those using display tactics (Evans, Pierotti, & Pilastro, 2003; Farr, 1980; Zimmerer & Kallman, 1989). Hence, sperm volume is an important predictor of success under sperm competition when multiple males compete for access to reproductive females (Bisazza, Vaccari, & Pilastro, 2001; Harcourt, Harvey, Larson, & Short, 1981; Harvey & Harcourt, 1984; Neff et al., 2003; Olsson, Madsen, & Shine, 1997; Stockley et al., 1997). When male–male competition occurs prior to fertilization (i.e., not as sperm competition), then other components of reproductive allocation such as display rate and investment in pigmentation will be more likely modes of reproductive allocation. Hence, in environments with low mortality risk, males should allocate energy to large body size, bright coloration, and high activity rate, but not necessarily to testes mass, as a means to compete for mating opportunities before fertilization (Evans & Magurran, 2001; Farr, 1980; Langerhans, Layman, & DeWitt, 2005; Zimmerer & Kallman, 1989).

Life history theory predicts that there should be differences in reproductive investment among populations when populations vary in expected mortality rates (Law, 1979; Michod, 1979). Specifically, males in high‐mortality environments should allocate more to reproduction compared to males in low‐mortality environments. Although several studies have tested these predictions in females (Johnson & Belk, 2001; Michl, Torok, Griffith, & Sheldon, 2002; Neff & Wahl, 2004; Reznick & Endler, 1982; Stockley et al., 1997), few have investigated how variation in mortality rates affects testes mass. Interestingly, those studies that have compared testes mass among species of livebearing fishes have found contradictory results. *Gambusia hubbsi* males have higher gonadosomatic indexes (GSIs) in high‐predation environments than in low‐predation environments (Riesch et al., 2013). In contrast, *Poecilia mexicana* males living in high‐mortality sulphidic environments have lower GSIs than those living in low‐mortality nonsulphidic environments (Riesch, Plath, & Schlupp, 2011). More recently, *Poecilia mexicana* and other related species exhibited no substantial differences in GSI across environments differing in mortality rates (Riesch et al., 2016). The results in *Gambusia hubbsi* (Riesch et al., 2013) are what we would expect according to life history theory: In high‐predation environments, males have less competition (lower density of conspecifics) prior to fertilization than males in low‐predation environments. Correspondingly, display rates are lower because males trade off sexual selection (opportunity to reproduce) with natural selection (due to predation; Godin, 1995; Langerhans et al., 2005; Luyten & Liley, 1985), and competition among males occurs after fertilization in the form of sperm competition rather than prior to copulation (Kawase et al., 2017; Stockley et al., 1997). Though studies have described testes mass as it fluctuates in various high‐mortality environments (Riesch et al., 2011), few studies have yet revealed how testes mass correlates with body mass among size classes and predation environments (Riesch et al., 2013, 2016).

**Figure 1:**
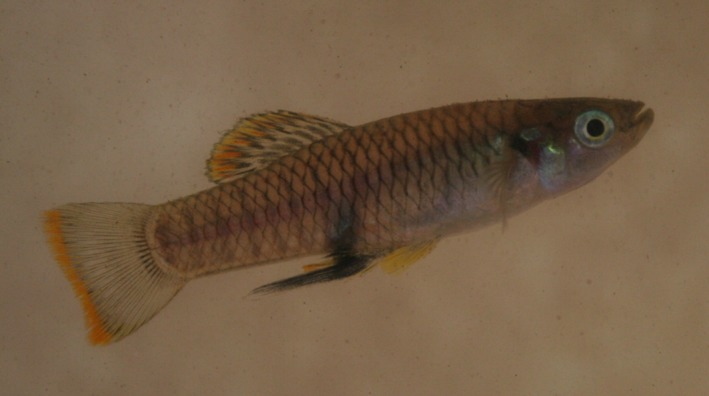
Male *Brachyrhaphis rhabdophora* from a high‐predation location—Rio Javilla Guanacaste Province, Costa Rica. Photograph by M.C. Belk

The livebearing fish *Brachyrhaphis rhabdophora* (Figure 1) is endemic to northwestern Costa Rica and is widely distributed in freshwater streams and rivers (Bussing, 1998; Johnson & Bagley, 2011). Adult male body size (i.e., somatic dry mass) within populations can vary by an order of magnitude (Johnson & Belk, 2001; Johnson & Bagley, 2011; Reznick, Meyer, & Frear, 1993), and male reproductive strategies vary with body size—large males rely on visual display and cooperative copulations, whereas small males are more likely to use sneak‐and‐thrust methods to gain forced copulations (Farr, 1989). In addition, mortality rates among populations of *B. rhabdophora* vary as a function of the presence or absence of predators (Johnson & Zuniga‐Vega, 2009). We use this system to test two hypotheses regarding male reproductive investment. First, we test for a hypoallometric relationship between testes mass and body mass among individuals within populations as predicted by sperm competition theory. Second, we test for differences in mean testes mass between populations from high‐predation environments and populations from low‐predation environments as predicted by life history theory.

## 2 METHODS

We collected male *B. rhabdophora* in the late dry season (late April, early May) of 1996 and 1997. Eight populations were collected in 1996, and one collection was added (population 33) in 1997. Although population 33 was collected one year later, it does not differ from the other populations in somatic mass of males, testes dry mass, or slope of the testes mass to body mass relationship. Overall results are the same whether population 33 is included in the analysis or not, suggesting no detectable year effect. The nine populations included here are a subset of those included in a previous study on female life history traits of *B. rhabdophora* in high‐predation and low‐predation environments (Johnson, 2001; Johnson & Belk, 2001). Four of our populations are from high‐predation environments and five are from low‐predation environments. Populations were chosen based on number of males in the collection, and each population was represented by about 30 individual males, for a total of 254 males analyzed (Table 1). Fish were collected with a hand‐held seine (1.3 × 5 m; 8 mm mesh size), preserved in ethyl alcohol in the field, and transported to the laboratory for further analysis. For details on methods and differences in predation environment among locations see Johnson and Belk (2001), and Johnson and Zuniga‐Vega (2009).

**Table 1:** Mean testes mass (*SD*), somatic mass (*SD*), standard length (*SD*), mean slope, and 95% CI of slope of the testes mass to body mass relationship of male *Brachyrhaphis rhabdophora* from five low‐predation populations and four high‐predation populations. Also included are means and slopes for combined samples

Predation environment	Population ID	*n* males	Mean somatic dry mass (*SD*) mg	Mean testes dry mass (*SD*) mg	Mean standard length (*SD*) mm	Slope of the testes to body mass relationship	Upper and lower 95% confidence interval	*R* ^2^
Low‐predation	21	36	66.7 (42.4)	1.6 (1.1)	25.2 (4.4)	0.28	−0.20 to 0.75	0.04
	19	46	98.1 (31.7)	1.2 (0.5)	28.0 (2.6)	0.64	0.35 to 0.94	0.31
	20	27	107.4 (52.4)	1.6 (0.9)	30.4 (4.7)	0.70	0.46 to 0.93	0.60
	27	19	61.3 (41.1)	0.6 (0.3)	25.0 (3.8)	0.23	−0.29 to 0.75	0.05
	33	27	69.3 (46.9)	1.3 (0.7)	24.1 (5.1)	0.67	0.41 to 0.93	0.52
	Combined	155	80.6 (20.7)	1.3 (0.4)	26.5 (2.6)	0.49	0.33 to 0.66	0.19
High‐predation	11	22	31.1 (17.5)	1.5 (1.2)	19.4 (2.9)	0.45	−0.32 to 1.23	0.07
	4	28	72.9 (38.7)	2.2 (1.8)	24.5 (3.8)	0.22	−0.42 to 0.86	0.02
	9	34	76.1 (42.4)	0.9 (0.6)	26.6 (4.3)	0.68	0.32 to 1.04	0.31
	23	15	40.7 (23.6)	0.6 (0.2)	22.0 (3.9)	0.39	−0.08 to 0.87	0.20
	Combined	99	55.2 (22.7)	1.3 (0.71)	23.1 (3.11)	0.38	0.19 to 0.73	0.09
All samples combined		254	69.3 (24.2)	1.3 (0.5)	25.0 (3.2)	0.44	0.24 to 0.68	0.14

For each individual, we measured dry mass of the testes and dry mass of the soma (hereafter testes mass and somatic mass). We dissected males, removing the digestive tract and the testes from the body. We measured somatic mass after testes and intestines had been removed (Tomkins & Simmons, 2002). Soma (minus the digestive tract) and testes were dried separately for 24 hr in a desiccation oven set at 55°C. Mass of dried tissue was measured to the nearest 0.1 mg. One person completed all dissections.

To test for allometry between testes mass and somatic mass, we used ordinary least squares regression on the data following a natural log transformation (Kilmer & Rodríguez, 2017). Testes mass was the response variable and somatic mass was the predictor variable. We tested for allometry, first within each collection location, second in combined samples pooled by predation environment, and third in combined samples pooled over all collection locations. In the analyses with combined samples, we included collection location as a random effect in our model such that variation among populations was not confounded with variation among individuals. To interpret these results, we assume a slope <1 indicates hypoallometry, a slope equal to 1 indicates isometry, and a slope >1 indicates hyperallometry.

To determine if testes mass differed among environments, we used a mixed model analysis of covariance (ANCOVA; Proc MIXED; SAS 9.3 SAS Institute, Cary, NC, USA: Littell, Milliken, Stroup, & Wolfinger, 1996). This model is the preferred model for testing for variation in reproductive allocation (Tomkins & Simmons, 2002). Again, we transformed testes mass and somatic mass using natural log to meet the assumptions of the statistical model. In these analyses, the dependent variable was testes mass, the independent variable was type of environment (high‐predation or low‐predation), and the covariate was somatic mass. We included somatic mass as a covariate to control for effects of body mass on testes mass across environments. This is similar to testing gonadosomatic index as the response variable and measure of relative testes size; however, use of testes mass as the response variable and somatic mass as a covariate is preferred (Tomkins & Simmons, 2002). We also included the interaction between predation environment and somatic mass in the model to test for variation in slope of the testes mass to somatic mass relationship between predation environments. Collection locations were treated as a random effect to avoid confounding location‐specific variation with variation between predation environments.

## 3 RESULTS

**Figure 2:**
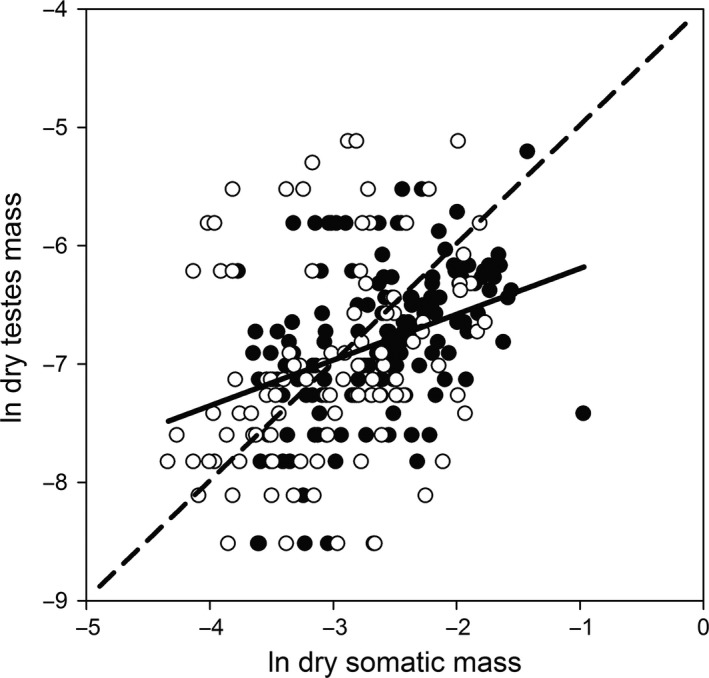
Relationship between ln‐transformed testes mass and ln‐transformed somatic mass for all samples combined. The best‐fit line and equation are from ordinary least squares regression with collection location as a random effect. Testes dry mass was hypoallometrically related to somatic dry mass. Regression equation: ln testes dry mass = 0.46 (ln somatic dry mass) – 5.6. *R* ^2^ = 0.15. Open circles represent individuals from high‐predation environments; closed circles represent individuals from low‐predation environments. The best‐fit line is solid, and the line of isometry is dashed

Within locations, testes mass was hypoallometrically related to somatic mass. All nine locations exhibited mean slope for the testes mass to somatic mass relationship >0 and <1 (Table 1; however, we note that in four of nine locations, there was a low goodness of fit with corresponding slopes no different from zero). Location‐specific slopes of the testes mass to somatic mass relationship ranged from 0.22 to 0.7 (Table 1). Thus, within populations, large males allocated proportionately less to testes mass than did small males. For samples combined across all high‐predation locations, samples combined across all low‐predation locations, and samples combined across both environments, testes mass was still hypoallometrically related to somatic mass (overall slope = 0.44, 95% confidence interval = 0.24 to 0.68; Table 1). The testes mass to somatic mass relationship is about 85% lower than expected for an isometric relationship. However, the overall relationship between testes mass and somatic mass exhibited high variability and poor goodness of fit (Table 1; Figure 2). On average, testes mass was 2.3% of somatic mass across high‐predation and low‐predation environments (95% confidence interval = 2.0% to 2.7%).

In contrast, predation environment was not a significant predictor of testes mass when adjusted for male body mass. However, the covariate somatic mass was significant, with smaller individuals having relatively larger testes mass than their larger counterparts. The interaction between predator environment and somatic mass was not significant (Table 2). Slope of the testes mass to body mass relationship for all populations combined in this analysis is 0.46, with a 95% confidence interval of 0.24–0.68, which compares favorably with the overall regression analysis above.

**Table 2:** Mixed model analysis of covariance results for variation in testes dry mass of male *Brachyrhaphis rhabdophora* in response to predation environment after adjusting for somatic dry mass

Source of variation	Degrees of freedom (num/den)	*F*‐value	*p*‐Value
Predation	1/62.7	0.01	0.9264
ln somatic dry mass (SDM)	1/250	41.38	<0.0001
ln SDM*predation	1/250	0.04	0.8338

## 4 DISCUSSION

Consistent with our expectations, in *B. rhabdophora* small males do allocate proportionally more energy to testes mass than do larger males. This result suggests that reproductive strategies associated with male body size explain at least some of the variation observed in testes mass in this species. We recognize that testes mass is only a part of reproductive investment made by males (Andersson, 1994). Testes mass could be the same between predation environments if other components of reproductive allocation such as behavior or activity rate are more plastic. However, our findings do appear to be consistent with what one would expect if sperm competition was occurring (Kawase et al., 2017; Stockley et al., 1997). Testes mass likely indicates how much sperm is available for reproduction, which in turn contributes to male reproductive success (Harcourt et al., 1981; Olsson et al., 1997; Stockley et al., 1997). In other words, the more sperm a male is able to produce, the greater chance that he will fertilize eggs and produce offspring (Harcourt et al., 1981; Olsson et al., 1997; Stockley et al., 1997). This is especially true of males engaging in sneak‐and‐thrust methods (Parker, 1970; Stockley et al., 1997). Our data support the idea that because successful fertilizations are low compared with the number of attempts for males that use sneak‐and‐thrust methods, they require a large store of sperm to gain reproductive success (Evans et al., 2003; Harcourt et al., 1981; Olsson et al., 1997; Stockley et al., 1997). An additional point about the relationship between testes mass and somatic mass is the wide range of variation in testes mass for a given body size and the consequent poor goodness of fit of the relationship as evidenced in Figure 2. This pattern of wide‐ranging variation is consistent with other studies on variation in testes mass (Tomkins & Simmons, 2002) and deserves further attention.

Curiously, female *B. rhabdophora* and several other species of poeciliids show hyperallometric patterns of reproductive allocation among females of different sizes (Belk & Tuckfield, 2010; Jones, 2014). Large *B. rhabdophora* females allocate proportionately more to reproduction than small females. Male *B. rhabdophora* differ in reproductive allocation from females, likely because their reproductive success relies on different factors. First, in contrast to females, males do not grow appreciably after they mature (Farr, 1989), so reduction in reproduction in small, reproductive males does not lead to greater reproductive capacity later in life. Second, males vary their mating strategy depending on their size relative to other males: small males tend to use sneak‐and‐thrust methods, and large males typically use display methods (Farr, 1989). At any size, females benefit from having more offspring and more room for developing offspring; however, they may tradeoff reproduction for growth when they are small to maximize future reproduction at a larger size, especially in low‐predation environments (Bashey, 2006, 2008; Bronikowski, Clark, Rodd, & Reznick, 2002; Clutton‐Brock, 1984; Gale et al., 2013; Johnson & Belk, 2001; Williams, 1966). On the other hand, small males, which primarily compete for reproductive success through postcopulation mechanisms (i.e., sperm competition), should benefit more from large testes than large males, which primarily compete for reproductive success through precopulation mechanisms (i.e., display rates, coloration; Farr, 1989). Thus, in *B. rhabdophora*, it follows that females should allocate to reproduction hyperallometrically across sizes while males allocate to reproduction hypoallometrically across sizes.

Interestingly, contrary to our expectations, we found no differences in testes mass between high‐ and low‐predation environments (Riesch et al., 2011) after adjusting for differences in body size. So, why does testes mass not vary between predator environments in *B. rhabdophora*? We offer two possible explanations. First, because male body size varies dramatically between high‐predation and low‐predation environments (about 30% difference based on body length, with males in high‐predation environments smaller, on average, than those in low‐predation environments; Johnson & Belk, 2001), and small males have proportionally larger testes than large males, then, on average, males in high‐predation environments do allocate more to testes mass compared to males in low‐predation environments. The difference in the average sizes between environments creates different proportions of average testes mass to average somatic mass such that the average GSI across all predator populations is 2.99% while the average GSI across all nonpredator populations is 1.90%. Thus, it may be that selection on body size at maturity as a consequence of differences in expected mortality rate (Law, 1979; Michod, 1979) facilitates differences in reproductive allocation as measured by testes mass.

A second alternative explanation for similar testes mass between predation environments, independent of body size effects, is that males may allocate to reproduction in areas other than testes mass. Both time and energy can be allocated toward external reproductive organs, display enhancers (e.g., coloration, feather length, fin size, antler or horn size) that increase the probability of success through female choice or male–male competition, and associated reproductive activities (e.g., courting, sneaking, mate guarding, etc.; Bonenfant, Pelletier, Garel, & Bergeron, 2009; Cummings & Gelineau‐Kattner, 2009; Heinsbroek et al., 2007). However, male poeciliids in high‐predation environments typically exhibit reduced coloration and reduced courting behavior compared to males in low‐predation environments (Endler, 1987; Godin & Briggs, 1996), suggesting that reproductive allocation via pathways other than testes mass may be constrained in high‐predation environments. Activity rates and patterns of allocating resources to reproductive activities are important determinants of reproductive success in many species (Gross, 1982; Neff et al., 2003; Toivanen, Rantala, & Suhonen, 2009; Wedell, Gage, & Parker, 2002; Ziege et al., 2009). In addition, sperm quality can vary independent of testes mass. In *Xiphophorus nigrensis*, testes mass is similar in sneaker and courting males, but sneaker males have sperm that is more viable and longer lived (Smith & Ryan, 2010). Hence, to fully understand male reproductive investment in *B. rhabdophora*, it may be necessary to consider differences in activity rates, size and coloration of male display organs, and sperm quality (Amrhein, Johannessen, Kristiansen, & Slagsvold, 2008; Bonenfant et al., 2009; Scantlebury, Waterman, & Bennett, 2008). This could be a promising area for future research.

## CONFLICT OF INTEREST

None declared.

## AUTHOR CONTRIBUTION

HNB and BHG participated in interpretation of data and drafting and revising article. JBJ and MCB participated in design and completion of experiment, analysis of data, interpretation of data, and drafting and revisions of article.

## DATA ACCESSIBILITY

Data available from the Dryad Digital Repository: https://doi.org/10.5061/dryad.nc57r1s.
